# The Use of Probiotics during Rearing of *Hermetia illucens*: Potential, Caveats, and Knowledge Gaps

**DOI:** 10.3390/microorganisms11020245

**Published:** 2023-01-18

**Authors:** Ellen Gorrens, Antoine Lecocq, Jeroen De Smet

**Affiliations:** 1 Research Group for Insect Production and Processing, Department of Microbial and Molecular Systems (M²S), KU Leuven, 2440 Geel, Belgium; 2 Department of Plant and Environmental Sciences, University of Copenhagen, 1871 Frederiksberg, Denmark

**Keywords:** bacterial supplementation, black soldier fly, microbiome engineering, microbiota, probiotics

## Abstract

Given the novelty of the industrial production of the edible insects sector, research has primarily focused on the zootechnical performances of black soldier fly larvae (BSFL) in response to different substrates and rearing conditions as a basis to optimize yield and quality. However recently, research has started to focus more on the associated microbes in the larval digestive system and their substrates and the effect of manipulating the composition of these communities on insect performance as a form of microbiome engineering. Here we present an overview of the existing literature on the use of microorganisms during rearing of the BSFL to optimize the productivity of this insect. These studies have had variable outcomes and potential explanations for this variation are offered to inspire future research that might lead to a better success rate for microbiome engineering in BSFL.

## 1. Introduction

The insect sector has grown significantly over the past years as insects are believed to have several advantages compared with traditional livestock species, such as the potential to upcycle various organic side streams and a lower environmental impact [1]. With already more than one billion euros of investments [2] and a market that is projected to expand at a compound annual growth rate (CAGR) of 12% from 2022 to 2027 [3], insect farming is clearly one of the new emerging agricultural sectors. Insects themselves can be considered as six-legged livestock in accordance with its legislative status. The European insect protein supply is expected to reach 1.2 million tons per year in 2025 produced by start-ups, medium-size companies and even highly automated industrial facilities [4,5].

In recent years, the black soldier fly (BSF) (*Hermetia illucens*) has gained enormous attention, mainly as protein source for feed and as a part of waste management strategies [6,7]. Thanks to their remarkable dietary plasticity, the black soldier fly larvae (BSFL) are capable of converting a wide variety of substrates into high quality insect biomass [1]. In nature, BSFL are found to thrive on decaying plant and animal matter, such as municipal waste and manure [8,9]. However, while they can grow on a wide range of substrates, this does not mean that they thrive on all of them. For example, feeding substrates that are high in lignocellulosic matter [10] and/or contain a high bacterial load [11] can reduce their growth performance drastically. Since insect producers rely on locally produced substrates, mainly by-products from the agri-food industry [12], research recently started to focus on strategies to engineer the microbiome and evaluate whether these strategies can enhance larval production.

Microbiome engineering is a research area that is recently gaining more and more traction due to advances in our ability to track and characterize microbial communities. In essence, microbiome engineering is the research field aiming to modify microbial communities to alter ecosystems of interest and/or to restore ecological balance [13,14]. This discipline of microbiome engineering has been around for some time, and is frequently applied to humans, animals, and plants. For animals, and thus for insect species, three main tools can be used to alter the gut microbiome: (i) enzymes, (ii) prebiotics, and/or (iii) probiotics [13]. Enzymes are proteins that can catalyze a reaction and are named according to the EC system updated by the International Union of Biochemistry and Molecular Biology (IUBMB) [15]. Next, prebiotics are defined as “nondigestible food ingredient that beneficially affect the host by selectively stimulating the growth and/or activity of one or a limited number of the bacterial species already resident in the colon, and thus attempt to improve host health” [16]. Finally, probiotics are characterized by FAO/WHO as “live microorganisms which when administered in adequate amounts confer a health benefit on the host” [17]. For other farm animals, the implementation of probiotics has led to a reduced occurrence of pathogens [18,19] and to enhanced lignocellulose degradation [20]. For instance, *Lactobacillus reuteri* protects poultry against *Salmonella* [21], and *Saccharomyces cerevisiae* addition to gnotobiotically reared lambs increased the fiber-degrading activities of cellulolytic bacteria [22]. The modes of action of these probiotics are mainly based on gut homeostasis, so knowledge on the composition and dynamics of the gut microbiota is fundamental for future (probiotic) research [18,23].

In that respect, the scientific knowledge on the gut microbiota of mass-reared BSFL has immensely expanded over the last few years. Just recently, a meta-analysis re-analyzed datasets with 16S ribosomal RNA gene sequence data of BSFL originating from 11 studies through the same bioinformatic pipeline. Regardless of the (experimental) conditions, members of the genera *Enterococcus*, *Morganella*, *Providencia,* and to a lesser extent *Klebsiella* and *Scrofimicrobium* were identified as core gut members [24], but their functions in the insect gut remain to be unraveled [24,25]. Despite this set of core genera, variation in the microbiota composition is influenced by feeding substrate, larval age, strain, location, and possibly other factors as well [24,26,27,28]. Recently, it was highlighted that the insect gut microbiota and its relationship with the conversion of side streams is a fruitful area for further work [1]. Remarkably, in 2018, probiotics in the field of industrially reared insects was considered almost virgin territory [23]. While now, just four years later, already three reviews have been published on the role of microorganisms in the context of insect mass production, summarizing the growing scientific knowledge [29,30,31]. The first one focused on microbes in mass-reared insects, focusing on their core composition, the influence of diet on their diversity, their relationship with reproduction, and impact of probiotic amendment [29]. The second one describes the role of probiotics in mass-produced insects with a focus on (i) pathogen reduction, (ii) typically used microorganisms as insect probiotics, and (iii) improved performance by probiotic administration [30]. The last one takes a look at the effects of microbes combined with mass-reared insects on their conversion efficiency, antibiotic degradation, pathogen inhibition, and odor removal with a focus on the core microbiota [31]. While these authors touched the subject of probiotic supplementation to BSFL, a more in-depth overview focusing solely on BSFL is warranted, given the leading role of this insect species in both industry and research these past years.

Therefore, this review draws on the recent literature pertaining to the impact of enzymes, prebiotics, and/or probiotics during rearing of BSFL to discuss the main findings on this topic. In addition, we aim to look at these attempts from a microbiome engineering angle to bring these concepts into the research on industrially produced insects and define caveats as well as knowledge gaps moving forward.

## 2. Search Procedure

To obtain an overview of the scientific studies dealing with strategies of microbiome engineering, a literature search using the following key words “black soldier fly”, “*Hermetia illucens*”, “microbiome engineering”, “enzymes”, “prebiotic”, “supplementation”, and “probiotic” was executed in Scopus and Pubmed in November 2022. Additionally, a cross-referencing approach was used to find other studies. To be included in the review, criteria were as follows: (i) publication in a peer-reviewed journal; (ii) publication in English; (iii) and research related to the introduction of enzymes, prebiotics and/or probiotics during rearing of *Hermetia illucens* aiming to enhance larval performances (pretreatment and fermentation procedures were excluded, nevertheless, these methods can be useful to improve larval bioconversion on recalcitrant streams [32]).

## 3. Search Results

The search resulted in a total of fifteen studies that were included in this review and described in Table 1. It also revealed that almost all relevant literature aims to influence the microbiome and thus insect performance via the supplementation of, suspected, beneficial microorganisms. Hence, this strategy of microbiome engineering is the focus. First, this review focuses on the origin and selection criteria for probiotic choice. Next, probiotic inoculation trials already performed are discussed focusing on the rearing conditions, inoculation of the probiotic, and their results. Finally, we describe hurdles and opportunities for future research and application potential in this field.

## 4. Origin of the Probiotics

In general, the origin of the probiotics used in the selected studies can be roughly divided into four categories: (i) gut isolates, (ii) isolates related to BSFL rearing, (iii) commercially available probiotics for other species, and (iv) other exogenous isolates (Table 1).

As a first source, most potential probiotics in the selected studies merely originate from the gut of BSFL (Table 1), indicating that microorganisms were isolated from the larval gut. The output of this isolation process depends on several factors, such as larval age, feeding substrate, and selection media. For instance, Mazza et al. [42] isolated probiotic candidates from the gut of the 5th instar larvae fed with 50% wheat bran and 50% corn flour, whereas Callegari et al. [38] isolated them from larvae fed with a nutritionally complete diet on which insect performance was high. The latter one assumed that it favors the development of a stable and healthy microbiota, increasing the chance to find an appropriate probiotic. In that study, gut dilutions were plated on different enriched media in order to select uricolytic and cellulolytic strains. In fact, culture-dependent studies on characterizing the BSFL microbiota have included various feeding substrates, several types of enriched, selective and general media, different cultivation strategies (direct plating and dilution-to-extinction cultivation), and incubation under oxic and anoxic conditions [25,38,46,48,49,50,51]. In these studies, some genera, such as *Proteus* and *Enterococcus*, were frequently cultured regardless of the set-up; however, some genera, for instance *Serratia* and *Rhodococcus*, were isolated exclusively in one particular study, indicating that the isolation process influences the obtained strain collection. After isolation of potential probiotics, some studies have characterized their strain collection on phenotypic properties potentially relevant for in vivo activity, such as enzyme activity, production of exopolysaccharides, and carbon utilization, to confer ecological advantages to the host [38,43,46]. Besides these metabolic screening tests, Savio et al. [30] listed a set of in vitro and in vivo screening techniques to characterize the probiotic potential of candidate strains for application in mass-reared insects environments such as tests focusing on adhesion properties. Remarkably, besides being a gut inhabitant, no additionally available information on the selection process and criteria to implement the particular microbe(s) is provided for most studies. This missing information is a limitation for future research to learn from past hurdles in the search for other candidates.

A second source for other studies was to augment a culture associated with *Hermetia illucens*, such as frass or substrate (Table 1). As substrates are an important determinant shaping the microbial community composition of BSFL [26,52], substrate-derived bacteria have been proposed as possible candidates [33,45]. For instance, the rearing residue at the end of a rearing cycle (consisting of unconsumed substrate, feces, and exuviae) was selected as an inoculum for the consecutive rearing cycle [45]. This technique is similar to backslopping in fermentation processes, in which a small part of a previously fermented batch is used as inoculum [53] and allows rearing facilities to easily generate its own inoculum [45]. While this might create opportunities for insect rearers in the future, caution is needed since unfavorable microorganisms can develop as well. Further, strains derived from the egg stadium might stimulate rearing performances [42]; however, the literature indicates that their relative importance is rather small in terms of larval performance and microbiota [54].

A third strategy is to evaluate commercially available commodities or well-known probiotics used for other species, such as for humans [34,36,40]. If beneficial results are achieved, exploitation of these strains is much easier since they are already commercially available. In line with this approach, probiotics that show promising results for other insect species might also be considered to be implemented during BSFL rearing.

Finally, some studies included strains from sources not related to *Hermetia illucens* or already commercially used. For instance, Rehman et al. [37] used a laboratory strain provided by another university and isolates from soil and pig manure fermentation. All strains exhibited cellulolytic activity tested on CMC plates. Next, oleaginous microbes, for instance *Rhodococcus rhodochrous*, have been opted to be of probiotic value due to their high fat content and versatile metabolism [40,41].

## 5. Set-up of Inoculation Trials for BSFL Rearing

**Figure 1:**
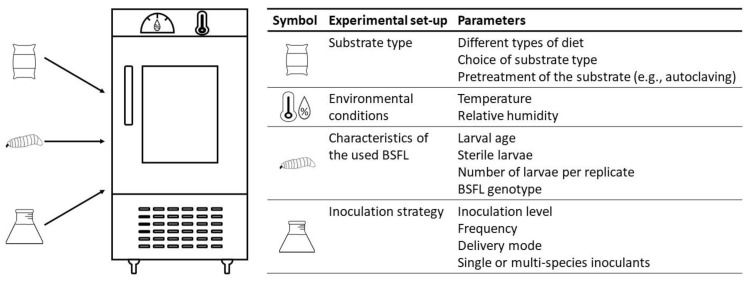
Overview of the set-up of an inoculation trial and a range of parameters that must be defined.

Once a potential probiotic is selected, it must be tested and a range of parameters must be defined. Unfortunately, these parameters are not consistent between the published studies, which hinders the proper comparison of the obtained results. Therefore, an overview is first provided of the selected rearing set-ups, summarized in Figure 1 before going to the actual results of different inoculation trials.

### 5.1. Substrate Type and Pretreatment

The diet that was provided to the neonates before inoculation was mainly bran-based and differed from most of the experimental diets (Table 1). This approach is consistent with standard rearing practices [55].

One can expect that there is little room for improvement for feeding substrates with already outstanding larval performances, such as chicken feed. On the other hand, diets consisting of lignocellulolytic biomass have demonstrated poor conversion into larval mass [32]. Thus, it is not unexpectedly that materials such as soybean curd residue [36], rice straw [34], and peanut shell powder [47] are included in studies to stimulate insect productivity. At the same time, as summarized in Table 1, many studies were performed on (chicken) manure as BSFL have been opted as a valuable solution for these types of waste to reduce ecological pollution [10]. However, its value as a waste management strategy is mainly theoretical in the European union, since manure is prohibited as a feeding substrate according to Regulation No. 1069/2009 [56]. Interestingly, feeding substrates were autoclaved in a few studies [41,43,47], probably to support colonization of the probiotics in the diets as this method would limit competition with other microorganisms. Yet, these sterilized substrates also resulted in a slower development of BSFL [23,47,57]. In fact, a small amount of non-sterilized substrate needed to be added to enable larval growth according to several industrial rearers [23]. Besides the loss of nutrients due to the autoclaving process, this slower growth on sterilized substrates indicates a prominent role of the gut microbiota in the productivity of the insect, for instance, by providing essential nutrients [23,57].

### 5.2. Environmental Conditions

With respect to the rearing environment, temperature (approximately 28 °C) and relative humidity (mostly around 60–70%) were in a small range, suitable for mass-rearing of BSFL (Table 1).

### 5.3. Characteristics of the Used BSFL

There is a great difference between the larval ages at the moment of supplementation (Table 1). For instance, Kooienga et al. [40] and Franks et al. [41] started their experiments with 11 day-old larvae, whereas in the study of Li et al. [43], sterile food waste was inoculated individually with six bacterial strains and served as feed for larvae directly after hatching to ascertain early colonization. Neonates might not have a fully developed microbiota [27], so it can be postulated that alteration of the microbiota is more feasible in younger larvae. In line with this, larvae originating from sterile eggs were used in some studies as well [46,47] to better understand the dynamics of possible probiotics. The use of these germ-free larvae probably promotes the gut colonization by the probiotic candidates.

A second parameter that varied was the number of larvae per replicate, ranging from 30 up to even a million larvae. As the environment varies between the different scale levels (other feed competition, temperature profile, moisture content, etc.), this can not only cause variability in conversion rate and larval efficiency [40], but the persistence of the probiotic after provision to the insect might change as well.

Another key parameter is the BSFL genotype used, since the BSF’s genetic background is known to impact BSFL performance, and perhaps the effect of specific probiotics as well [28,58]. The so-called Wuhan strain has been used most frequently in the studies presented in Table 1 [34,35,36,37,42,46].

### 5.4. Inoculation Strategy

The inoculation level of the administered bacteria was around 6 log cfu/g diet in most studies, with 8 log cfu/g as the highest mentioned level (Table 1). A bacterial suspension or pellet obtained from an overnight culture was mostly added and mixed in the substrate. Specifically for *Bifidobacterium breve*, the only strictly anaerobic bacterium from Table 1, the Gainesville diet was placed in an anaerobic chamber and then it was inoculated with the probiotic and left for colonization before larvae were added [40]. Supplementation with the particular microbe occurred in most studies only at one time point as this is economically more feasible for companies. Nevertheless, inoculating multiple times has been executed as well, probably increasing the chance of gut colonization.

Most inoculation trials have tested probiotics individually supplemented to the rearing system, whereas some have added polybacterial inoculants.

## 6. Probiotic Inoculation Trials for BSFL Rearing

Although most studies present in Table 1 achieved promising results with the microorganism(s) tested, there were also probiotic candidates that had no or even detrimental effect. To discuss these probiotic inoculation trials results, a division based on the classes of the candidate probiotic was made.

### 6.1. Potential of Bacilli

A particular set of studies present in Table 1 has focused on the effect of *Bacillus* species on BSF fitness. This genus has been used as probiotics in other mass-reared insect species as well [30]. In particular, *Bacillus subtilis* strains, mainly derived from the larval gut, have been suggested as a promising candidate for manure reduction. Larvae fed manure supplemented with these targeted bacteria showed an increase in waste reduction rate, compared with BSFL without bacteria inoculation, of 8.8% [42], 13.4% [35], and 16.2% (only defined as *Bacillus* strain, isolated from pig manure fermentation) [37]. Dry larval mass gain ranged between 15.9% and 58.7% [33,35,37,42]. Moreover, these experiments yielded an accelerated larval development, higher survival rate, and lower feed conversion ratio. Interestingly, while a *B*. *subtilis* strain from the larval gut had positive effects in the study of Mazza et al. [42], the addition of chicken manure with another *B*. *subtilis* strain, isolated from the eggs, did not enhance larval development in the same study. As functional traits of closely related strains can differ considerably [59], the beneficial effects of Bacilli might well be strain-dependent. Hence, in vivo validation of a possible probiotic candidate remains necessary, and one cannot just assume beneficial effects.

In another study, supplementation of a *Bacillus licheniformis* strain isolated from the larval gut also resulted in higher larval weight (about 20%) and growth rate on a nutritionally poor fruit diet [38]. Finally, another species of this genus, *Bacillus velezensis* EEAM 10B, isolated from the larval gut, was shown when administered to BSFL fed food waste mixed with peanut shell powder, to elevate the substrate conversion rate by 5%, the average dry weight by 0.13 g/larva and the protein content of the larvae by 8%, while the survival rate and the substrate consumption rate were not improved [47]. Most likely these effects were due to the colonization of the gut by the supplemented bacteria, as the relative abundance of *Bacillus* in the gut increased by 12% when *B*. *velezensis* was added and the protease, amylase, cellulase, and lipase activity in the midgut were higher. This indicates that the gut was colonized by the target bacteria and the abundance of other intestinal microbes was affected [47]. Another study exploring the colonization potential by probiotic candidates used neonate larvae provided autoclaved food waste supplemented individually with six different candidates to track their early colonization. Nonetheless, the amount of these bacteria in the larval intestine after 24 h during starvation was only increased for one target bacteria, being *Lysinibacillus sphaericus* [43]. Of these six candidates, only *Lysinibacillus* was a spore former. This characteristic might benefit the colonization of the gut, since these endospores have a higher survival rate through the acidic gut passage [60]. In fact, probiotics must pass the lumen of the middle midgut with a low pH around 2 and high lysozyme activity to arrive in the posterior midgut with a pH of approximately 8.5 [61].

In another study, a well-known probiotic *Lactobacillus buchneri* was successfully added to co-convert soybean curd residue. This supplementation increased among other things, the survival rate, the larval weight, bioconversion rate, and crude protein and fat content [36].

Additionally, a *Pediococcus pentosaceus* strain was tested for BSFL rearing. This strain was isolated from the guts of *Tenebrio molitor* larvae and achieved beneficial effects when it was provided to that insect [62]. The strain was added individually as a bacterial suspension or freeze-dried powder at three time points (day 0 when eggs were added, day 4 and day 8) to the substrate with an inoculation level of 8.7 log cfu/g (DM). Two diets (chicken starter mash and wheat bran) were tested until the larvae were harvested at day 17. Survival, growth rate, bioconversion, and waste reduction, number of prepupae and dry matter were monitored, but no promising results were achieved [63]. This outcome indicates that the impact of a probiotic is likely host-specific, which is not very surprising since probiotic candidates are mainly identified as members of the core microbiota of the insect itself [30].

### 6.2. Potential of Gammaproteobacteria

Gammaproteobacteria are another class of bacteria that have been often added to BSFL. For instance, Li et al. [46] augmented separately six isolates retrieved from the larval gut to sterile larvae. Of these isolates, *Providencia* sp., *Citrobacter* sp., *Klebsiella* sp., and *Proteus* sp. belong to Gammaproteobacteria (whereas *Dysgonomonas* sp. and *Ochrobactrum* sp. not). Except for *Proteus* sp., the inoculation of these bacteria resulted in BSF weight gains (even a 10% increase for *Klebsiella* sp. larvae group) and increased development compared with the germ-free control. Via PCR, it was shown that the larval gut was colonized by these six bacteria individually, as is probably more feasible in sterile larvae compared with ‘normal’ larvae as already touched upon. Surprisingly, the addition of *Proteus* sp. to these germ-free larvae resulted in a decrease in larval weight and a prolonged BSF life cycle. In comparison, when *Proteus mirabilis* isolated from the egg surface was added to chicken manure, the larval weight, conversion rate, and manure reduction rate were improved [42].

In another study, 193 isolates from the larval gut were screened and in addition to a *Bacillus licheniformis* strain, a *Stenotrophomonas maltophilia* HI121 was selected for an in vivo test thanks to its diverse metabolic activity profile: it was able to digest casein, to release ammonia, to degrade organic phosphorus and pectin, and to exert lipase activity. Nevertheless, the supplementation did not generate positive results [38]. The authors stated that there is a need to experimentally verify whether these probiotic actions indeed take place in vivo. Additionally, it can be questioned whether these metabolic actions are relevant for the rearing system used since, for instance, lipase activity might not be required for a fruit-based diet. Remarkably, in the same study an *Escherichia coli* strain not identified as a member of this specific BSFL gut community, thus used as a control strain, led to an increase in larval weight and growth, similar to the addition of *Bacillus licheniformis* previously discussed in Section 6.1 [38].

### 6.3. Potential of Microbes from Other Classes

Next to Bacilli and Gammaproteobacteria, other classes of microbes have also been opted for as potential probiotic candidates. In the class of Actinomycetia, the oleaginous microbes *Rhodococcus rhodochrous* and *Arthrobacter* sp., have been tested. At a bench scale, daily feed supplementation with a fat-rich *Rhodococcus rhodochrous* strain to the (autoclaved) Gainesville diet resulted in higher waste conversion rates, a near doubling in larval weight and differences in fatty acid composition and protein expression profiles [41]. Moreover, the daily supplementation of an *Arthrobacter* strain to 300 larvae resulted in higher larval weight and lower waste:larva ratio [40]. At an industrial scale using approximately 10,000 BSFL, the Gainesville diet was inoculated once with either *Arthrobacter* or *Rhodococcus rhodochrous*. Initially, at day 3 and 6 this resulted in higher larval weights, but at day 10 the control larvae weighted 6.3% and 12.0% more than the *Arthrobacter* and *Rhodococcus* group, respectively, possibly due to pupation in the augmented groups [40].

The supplementation of *Bifidobacterium breve*, a well-characterized human probiotic, during rearing of BSFL had an inverse effect on larval growth and waste conversion. Moreover, these larvae appeared “discolored, slow, covered in a sticky exudate and overall unhealthy” and yielded larvae being 50% less in final weight [40]. In addition, the incorporation of live yeast, *Saccharomyces cerevisiae*, did not alter the fatty acid composition of BSFL, even though it can produce unsaturated fatty acids and the proportion of unsaturated fatty acids was increased in the substrate [44]. As black soldier fly larvae mainly consist of saturated lauric acid (C12:0) [64], modulation of this fatty acid profile can be beneficial when the larvae are fed to mammals [65] and aquaculture [66,67,68]. Additionally, yeast, which is the most common probiotic for ruminants, can scavenge oxygen, thereby creating more anaerobic conditions. This can generate more favorable conditions for anaerobic communities [18].

### 6.4. Potential of Polybacterial Inoculants

Next to the addition of probiotic candidates individually to the rearing system, a combination of two or even more bacteria was applied as well, thus exploring potential complementary actions, for instance for lignocellulolytic degradation. In the study of Zheng et al. [34], (hemi)cellulose, lignin, and protein conversion rates were increased with the aid of a commercial commodity containing both microbes and enzymes. The same product was used in another study where the highest inoculation level resulted in higher growth rate, body mass, and protein yield [39]. In fact, enzyme pre-treatment of lettuce–cabbage waste resulted in an improved biomass conversion efficiency by 22% [69].

*Stenotrophomonas maltophilia*, which showed no positive results when added as a monobacterial inoculum (see Section 6.2), was added together as a polybacterial inoculant with *Bacillus licheniformis*, larval and pupal weight were increased again. This can be an indication that only the latter one executed beneficial effects [38]. In contrast, when mixtures of different bacteria (*Bacillus subtilis*, *Kocuria marina*, *Lysinibacillus boronilerans* and *Proteus mirabilis*) with individually good results were combined, the outcomes were, surprisingly, dependent on the ratio of these strains [42]. To decipher the exact mechanisms resulting in these varying results, more research is needed.

Finally, the substrate-derived inoculum, both from tomato pomace and white wine pomace, did not yield improved performances or alteration of the gut microbiota [45].

## 7. Hurdles in the Evaluation of a Potential Probiotic

All these studies show that there has been a rapid increase in research exploiting potential probiotics to benefit insect productivity over these past years. Despite this growing interest, there are some major hurdles to overcome and opportunities to explore in further research and valorization, which is discussed in these last two sections.

### 7.1. Confirming the Establishment of Probiotic in BSFL

As in most studies the microbe was not monitored once added, it seems possible that the conditions to thrive in the rearing system were not favorable for the candidate organisms, and thereby, these microbes might not have survived their introduction in the feeding substrate, which might result in false-negative results. For instance, strictly or facultatively anaerobic microbes (which are abundantly present in the BSFL gut) are exposed to oxygen when added to the feeding substrate, probably affecting the viability of the strain. Since survivability of the probiotic throughout the gut was identified as a key factor for optimal efficacy [22,43], this oxygen preference must be taken into account. In order to deliver bacteria in a viable form and to bypass this oxygen sensitivity, the delivery mode might need to be changed, for instance by adding lyophilized or protected cells [14,70]. In addition, to confirm the establishment and the stability of this probiotic candidate, monitoring of the probiotic is essential for future inoculation trials. Possible strategies are to include DNA or RNA-based techniques [40], to genetically modify the probiotic for detection on plates [11,71], or to fluorescently label the probiotic [72].

Of these methods, only quantitative PCR (qPCR) has been used in the existing literature to detect *Arthrobacter* in a small-scale experiment and both *Arthrobacter* and *Rhodococcus rhodochrous* in an industrial scale experiment [40]. At benchtop scale, *Arthrobacter* was indeed detected via qPCR in the larval intestine over a period of 10 days after daily supplementation. As this fat-rich microbe was supplemented daily and bacteria can serve as a food source for larvae [73], the detection via qPCR does not prove in this case that *Arthrobacter* can function as a probiotic or was just present as ‘food’ in the gut, as further explained. When *Arthrobacter* or *Rhodococcus rhodochrous* were only added once at the industrial scale, qPCR revealed a decrease in the presence of both microbes, with *Rhodococcus* only above the detection level at day 3. These results suggest that these candidates could not colonize the gut and act as a probiotic in the larvae. An important attention point when using DNA-based techniques to confirm the presence of viable microbes is that they are, without additional processing steps, not capable of distinguishing between dead and living cells [74].

### 7.2. Differentiating between a Nutritional or a Probiotic Effect for the Inoculated Bacteria

In these inoculation trials, it is important to distinguish the role of the probiotic as solely a nutrient source or as an active contributor to insect physiology by establishing interactions with the insect. For example, it is likely that the nutritional value of the Gainesville diet was improved by daily supplementation of the fat-rich microbe *Rhodococcus rhodochrous* [41], regardless of whether it could act as a probiotic, thus BSFL can digest it to thrive more easily and accumulate energy. In another study, the addition of *Escherichia coli* also led to an increase in larval weight. The authors hypothesized that this supplementation improved the nutritional quality of the diet and thereby improved the larval performance [38]. Further, when a commercial commodity consisting of both enzymes and microbes was tested, the enzymes, being cellulase, lipase, protease, and amylase, could help degrade (lignocellulosic) materials thereby providing nutrition to the BSFL, regardless of the possible contributing effects of the microbes [34].

To tackle this issue, an additional control group where the diet is augmented with the inactivated probiotic, for instance by autoclaving, should be included in the experimental set-up [45,62]. If such control group is not added and promising results are achieved, the researchers must consider to monitor the nutritional value of the feeding substrate compared with the control during inoculation trials. As such, modulation of the nutrient composition of BSFL has been achieved by the inclusion of algae in order to increase the omega-3 polyunsaturated fatty acid content, which is especially interesting for the aquatic industry [66,67,68]. As these algae probably only act as a nutrient source and are not naturally present in the larval gut, these studies were not included in this review.

### 7.3. Impact of the Inoculation Strategy

The inoculation strategy comprises the dose, frequency, delivery mode, and growth stage (stationary or exponential growth) of the microbes and whether single or multi-species inoculants were introduced to the rearing system. For inoculation trials with poor results, alterations to the inoculation strategy might result in more promising outcomes.

A possible modification is to adjust the inoculation level by either adding a higher or a lower dose [14]. Adding more probiotic cells might result in a sufficient amount of inoculum that can reach the gut, while adding less might result in less competition between the gut members, for instance, for food [14,39]. Further, inoculating the substrate on a more frequent basis (for instance daily as executed by [40], but not discussed by the authors) could probably enhance gut colonization more than a one-off inoculation [14], though it also increases the risk of observing nutritional effects of the continuous supplementation. As already discussed in Section 7.1, the form in which the probiotic is offered to the larvae can affect the outcome of the inoculation trial, and it might be advised to use lyophilized or protected cells or if possible spores, which are more likely to persist the passage through the middle midgut [14,60,70]. Finally, for multi-bacterial inoculants, the outcomes were dependent on the candidates and on the ratio of the candidates [38,42]. The precise mechanisms of these multi-species inoculants remain to be elucidated. In most studies in related research areas, the outcomes of the multistrain and/or multispecies formulations were superior to monostrain probiotics. Among other things, synergistic effects of the species, greater divergency, and symbiosis among strains have been mentioned as possible mechanisms underlying the enhanced effects [75,76]. Antagonistic plate-assays can shed a light on the interactions between the microbes and were used in these studies to find an explanation why no beneficial effect was observed constantly [38]. In the future, microbiome transfer might be an option in accordance with fecal microbiota transplantation performed in humans [13,77].

## 8. Remaining Knowledge Gaps for BSFL Probiotics

### 8.1. Expanding Our Fundamental Knowledge on the Role of Microbes in BSFL

The first challenge is to unravel the mechanisms resulting in enhanced BSFL performances when companion microbes are augmented, since those are unclear [42]. Probiotics are postulated to deliver nutrients and growth factors [10,18] to facilitate the digestion of recalcitrant (such as lignocellulosic) materials and make these more accessible for the larvae [40,41], to balance or colonize the gut and thereby alter the gut’s microbial composition [18,40,47], to affect the initial environment and thereby impact the proliferation of certain bacteria [40], to protect the larvae from pathogens [78], to influence the immune response of the host [18], and/or to affect metabolism processes such as amino acid synthesis and vitamin metabolism [47]. If future studies on probiotics in the context of industrial insect rearing can map which of these complex modes of action are predominant or most effective in BSFL, research can focus on isolates with such abilities. Pei et al. [47] performed, for instance, vitamin backfill assays, which suggested that the microbe being tested as a probiotic, i.e., *Bacillus velezensis*, was responsible for delivering riboflavin to BSFL. Therefore, expanding knowledge on the mechanisms of probiotics in BSFL can lead to (i) the selection of already described microorganisms with the desired properties and (ii) the alteration of the isolation process and/or in vitro assays to enhance selection of promising candidates for in vivo tests. As previously summarized, provision of microbes during rearing can result in no or even a negative impact, so probiotic selection is an important issue for future research. In line with this, an upcoming methodology to unravel or validate functions of specific microorganisms is to inoculate them to sterile BSFL originating from sterile eggs, thereby constructing a mono- or multi-bacterial intestinal model [47,54]. Protocols to produce sterile eggs have been established during the last years [46,54,57,79]. These developments can support the probiotic selection; however, since the gut is a collaborative environment, interactions between gut habitants are not observed using a sterile BSFL model which limits the use of this approach [47].

### 8.2. Correlation between Lab-Scale Research and Industrial Scale Production

Another knowledge gap in this field is that most studies have been conducted at laboratory scale and on only one feeding substrate. It is questionable whether the same effects would be achieved at an industrial scale since, e.g., nutrient availability, moisture content, and heat flow differ from the small scale [40]. For instance, results of the supplementation of *Arthrobacter* AK19 at benchtop and industrial scale differ in terms of mean larval weight, waste:larva ratio, and gut colonization; however, comparable results were observed as well [40]. When beneficial microorganisms yield positive results in a particular substrate, extrapolation of these results to other feeding substrates is also not straightforward. Therefore, the effectiveness of each promising microbe should be validated on a larger scale (pilot or industrial level) and on other diets. Next to the feeding substrate, the effect of larval strain, rearing conditions, and other (a)biotic factors on the probiotic application should be investigated as well.

The interest of the insect sector in the probiotic application is currently unknown. For other farm animals, a robust feed additive sector has been established, commercializing different products, such as enzymes, vitamin mixture, and probiotics [80]. Exploitation of an efficient probiotic for BSFL opens new doors for this sector as they can enter the growing market of industrial insect production. However, to the authors knowledge, no information exists on the commercial willingness of the insect sector to use such products (e.g., conditions for use, maximal cost companies are willing to pay, and return on investment expected). Input of the insect sector is crucial before commercialization of a promising probiotic is even possible. Further, their input can help research to focus on those feeding streams and preferred benefits (e.g., improved feed conversion ratio, higher survival rate) most relevant for the insect sector. For instance, manure has been selected as a feeding substrate in many studies dealing with probiotic addition (Table 1); however, this feeding substrate is not allowed in the European Union, which limits the exploitation potential of such probiotics.

### 8.3. Expanding towards Other Probiotic Effects Than Growth Improvement

The studies presented in this review have merely focused on the insect growth-promoting effects of probiotics. On the other hand, microorganisms can be used for biological control as the gut microbiota plays a pivotal role in preventing colonization by invading pathogens [18,78]. For instance, supplementation of lactobacilli to honeybees decreased pathogen load during a *Paenibacillus larvae* infection [81]. Due to the intensive way of insect mass-rearing, outbreaks caused by entomopathogens (i.e., insect pathogens) are likely to occur in production units [82]. Here, biological control agents against entomopathogens of BSFL can be developed as a tool to reduce those risks. To date, such research has barely been touched upon to the authors knowledge. This is probably because no disease outbreak has been reported yet in BSFL rearing facilities in contrast to other mass-reared species [83]. Nevertheless, there is a scarcity of knowledge on the susceptibility of *Hermetia illucens* for different types of insect pathogens: infection with the fungus *Beauveria bassiana* has been demonstrated for in vivo conditions and injection assays with different microbes resulted in between 0% and up to 100% lethality [49,84]. Additionally, a distinction needs to be made between entomopathogens and food/human pathogens. Infection with the latter one can be harmless for the insect itself, while it might be a threat to food safety. For instance, when BSFL were inoculated with *Staphylococcus aureus* or *Salmonella* species, no effect on the larval growth was observed [11,71]. Probiotics for BSFL to sustain low pathogen load in the digestive system can be the focus of future research. For instance, 15 BSFL gut isolates of the collection of Tegtmeier et al. [51] showed ability to inhibit *Escherichia coli* and/or *Pseudomonas aeruginosa* and/or *S*. *aureus*, and *Trichosporon* strains associated with *Hermetia illucens*, have shown great in vitro activity against *S*. *aureus* [11] and are thus already suggested for probiotic application [51]. In addition, these isolates can lead to the discovery of new antimicrobials [85].

Finally, bacteriophages, viruses that infect and kill bacteria, might be an interesting tool for microbiome engineering as well. An *Escherichia* phage has already been isolated from BSFL and its induction was dependent on the substrate [86]. Each bacteriophage infects only a very specific host microbe and can thus be used as a high-specific microbiome engineering tool to target a specific bacterium and to alter the community composition [77]. It is clear that considerably more work is needed to tackle all these issues.

## 9. Conclusions

In the last years, more work has been performed on microbe supplementation within the BSFL rearing system to promote key insect traits. To identify appropriate microbes for probiotic application, different search strategies have been executed, in which isolates associated with the larval gut have been screened the most, with varying results.

In rearing trials, among other things, increased survival rate, substrate conversion rate, and larval weight have been observed when one or more probiotics were added to the diet. Nevertheless, supplementation of the feeding substrate with one or a combination of multiple microbial species led at the same time to no or even detrimental effects. Directing research efforts towards improving the understanding of the insect–microbe dynamics, the necessary working conditions for probiotics and the selection of appropriate candidates is crucial. Indeed, at the moment it remains impossible to predict based on species, enzyme activity, or any other parameter if a microbe will have a beneficial effect. The publication of both negative and positive results from probiotic analysis studies in BSFL is key to improving such predictions. In future inoculation trials, the experimental set-up should be thoroughly designed dependent on the aim of the study, for instance, when the survival rate needs to be improved, young larvae can be inoculated as they are the most vulnerable, but when cellulase-producing probiotics are selected, these probiotics can be added to a cellulose-rich substrate after the nursing phase. Additionally, a control group consisting of inactivated probiotics should be included as well.

It can be concluded that while it is too early to conclusively say how much gain can be made with probiotics in BSFL rearing, the current state-of-the-art does hint at an important role to be played by microbes to boost BSFL performance, especially on less digestible and/or poorer diets, and this warrants further (fundamental) research on this topic to understand the underlying mechanisms for better probiotic candidate selection.

## Figures and Tables

**Table 1:** Overview of the fifteen selected studies with details about their set-up and results.

Experimental Set-Up								Way of Inoculation				Results Compared with the Control Group ^1^								
Probiotic Candidate	Origin of the Probiotic	Substrate before Inoculation	Substrate after Inoculation	Temp. (°C) *	RH (%) *	Larval Age at Inoculation (Days)	# of BSFL per Replicate *	Suspension	Pellet	Single Inoculation	Concentration	Weight of BSFL	Protein Content	Fat Content	Conversion Rate	Duration of the Rearing Cycle	Survival Rate	Growth Rate	Gut Colonization	Reference
*Bacillus subtilis* S15	Larval gut	Artificial grain-based diet	Chicken manure	27–30	80 ± 8	4	100	✓		✓	6 log cfu/g	+				+	↔			[33]
*Bacillus subtilis* S16												+				+	↔			
*Bacillus subtilis* S19												+				+	+			
*Bacillus natto* D1	Diet fed to BSFL											+				+	↔			
Rid-X	Product with microbes and enzymes	Standard colony diet	Mixture of restaurant waste and rice straw	27	70	6	≈2000				0.05 to 0.5% (*w*/*w*)			+	+					[34]
*Bacillus subtilis*	Larval gut	Bran-based diet	Chicken manure	25–45	NA	6	One million	✓		✓	6 log cfu/g	+			+					[35]
*Lactobacillus* *buchneri* L3-9	Laboratory strain	Standard fly larvae diet	Soybean curd residue	27	70	6	1000	✓		✓	6 log cfu/g	+	+	+	+	+	+			[36]
*Paenibacillus plymyxa* strain KMZ (R_1_)	Soil	Bran-based diet	Dairy and chicken manure in a 2:3 ratio	27	60–70	6	1000	✓		✓	6 log cfu/g	+			+	↔	+			[37]
“ZRO_2_” (R_2_)	Laboratory strain											+			+	↔	+			
*Bacillus* strain (R_3_)	Pig manure fermentation											+			+	↔	+			
*Bacillus* strain (R_4_)												+			+	↔	+			
*Bacillus* strain (R_5_)												+			+	↔	+			
*Bacillus* strain (R_6_)												+			+	+	+			
*Bacillus* *licheniformis* HI169	Larval gut	Nutritionally poor diet composed of apple (1/3), pear (1/3) and orange (1/3)	Nutritionally poor diet composed of apple (1/3), pear (1/3) and orange (1/3)	25	60–65	9	150	✓		✓	≈7 log cfu/g	+						+		[38]
*Stenotrophomonas maltophilia* HI121												↔						↔		
Combination of the two above												+						↔		
*Escherichia coli* DH5α	Laboratory strain											+						+		
Rid-X	Product with microbes and enzymes	Fresh coconut endosperm waste medium	Coconut endosperm waste	NA	60–65	6	20			✓	0.02%	−	+	−	+	+		−		[39]
											0.10%	↔	+	+	↔	↔		+		
											0.50%	−	+	−	+	↔		−		
											2.50%	+	+	↔	+	↔		+		
*Bifidobacterium breve*	NA	Spent grain diet	Gainesville diet	Room temperature	NA	11	100		✓		≈6 log cfu/g	−			−					[40]
*Arthrobacter* AK19							300		✓		≈5 log cfu/g	+			+			+		
				NA			≈10,000		✓	✓	≈3 log cfu/g	↔			↔			+		
*Rhodococcus* *rhodochorus* 21198							≈10,000		✓	✓	≈3 log cfu/g	↔			↔			+		
*Rhodococcus* *rhodochrous* 21198	NA	Gainesville diet	Gainesville diet	28	60	11	300		✓		≈5 log cfu/g	+			+			+		[41]
			Autoclaved Gainesville diet									+			+			+		
*Bacillus subtilis* (A)	Larval gut	Wheat bran with 75% water content	Chicken manure	28	60–70	6	500 ^2^	✓		✓	6 log cfu/g	+			+					[42]
*Kocuria marina* (B)	Egg surface											+			+					
*Lysinibacillus* *boronitolerans* (C)												+			↔					
*Proteus mirabilis* (D)												+			+					
*Micrococcus luteus*												+			↔					
*Enterococcus* *faecalis*												+			↔					
*Sporosarcina* *koreensis*												↔								
*Gordonia sihwensis*												+			↔					
*Enterobacter* spp.												↔								
*Bacillus subtilis*												↔								
Polybacteria community (A; B; C; D) at different ratios	See above											Results vary between the different ratios.								
*Lysinibacillus sphaericus*	Larval gut	/	Autoclaved food waste	28	60–70	Directly after hatching	Starting from 2 g of eggs	✓		✓	NA								+	[43]
*Enterococcus* *faecalis*																			↔	
*Proteus mirabilis*																			↔	
*Citrobacter freundii*																			↔	
*Pseudocitrobacter faecalis*																			↔	
*Pseudocitrobacter anthropi*																			↔	
*Saccaromyces* *cerevisiae* Meyen *ex* E.C. Hansen		Apple (55%) and beer malt (15%)-based	Yeast liquid from beer brewery waste	30	30	Directly after hatching	1000	✓			NA								+	[44]
Tomato pomace-derived inoculum	Rearing residue	Poultry feed	Tomato pomace	28	NA	To 0.8–1.1 mg DM/larva	≈200	✓		✓	7.5 log cfu/g DM	−								[45]
White wine pomace-derived inoculum			White wine pomace								8.0 log cfu/g DM	↔								
*Providencia* sp.	Larval gut	Artificial diet (25% corn meal, 75% wheat bran)	Sterilized artificial diet (25% corn meal, 75% wheat bran)	28	70	Neonate larvae (newly hatched)	40 germ-free larvae ^2^	✓			NA	+				+			+	[46]
*Citrobacter* sp.												+				+			+	
*Klebsiella* sp.												+				+			+	
*Dysgonomonas* sp.												+				+			+	
*Ochrobactrum* sp.												+				+			+	
*Proteus* sp.												−				−			+	
*Bacillus velezensis* EEAM 10B	Larval gut	Wheat bran	Food waste mixed with ±10% peanut shell powder	30	70	3rd instar	NA	NA			NA	+	+	↔			↔		+	[47]
		Brain heart infusion medium	Autoclaved food waste	37	NA	NA	30 sterile larvae ^2^	NA			NA	+					+			

* The following abbreviations were used: Temp.: temperature; RH: relative humidity; #: number. ^1^ The main results have been selected, so this list is not complete. Explanation symbols: ‘+’ means there is a positive impact, ‘↔’ no impact, and ‘−’ a negative impact. ^2^ Compared with a sterile control group.

## Data Availability

Not applicable.
